# Supplementary material for: Development of Simultaneous Determination Method of Pesticide High Toxic Metabolite 3,4-Dichloroaniline and 3,5 Dichloroaniline in Chives Using HPLC-MS/MS
Source: Foods. 2023 Jul 28;12(15):2875. doi: 10.3390/foods12152875 (PMC10417142; doi:10.3390/foods12152875)
Supplement: Supplementary file 1 [file foods-12-02875-s001.zip › foods-2520247-supplementary.pdf]

Supplementary Materials

Table S1. The primary physico-chemical properties of 3, 4-DCA and 3, 5-DCA.

Compound name	Molecule	Molecular formula	Molecular weight	Melting point (°C)	Boiling point (°C)	Solubility in water (g/L)
3,4-DCA	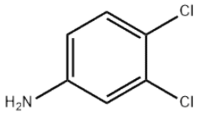	$C_6H_5Cl_2N$	162.02	69-71	272	0.6
3,5-DCA	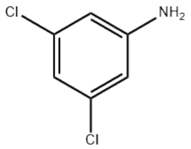	$C_6H_5Cl_2N$	162.02	46-52	260	0.6

Table S2. The MS/MS parameters of two DCAs.

Compound	Cone voltage (V)	Precursor Ion /(m/z)	Quantitative ion /(m/z)	Collision energy /eV	Qualitative ion /(m/z)	Collision energy /eV
3,4-DCA	115	163.03	128.0	20	75.1	60
3,5-DCA	115	163.03	128.0	20	75.1	60

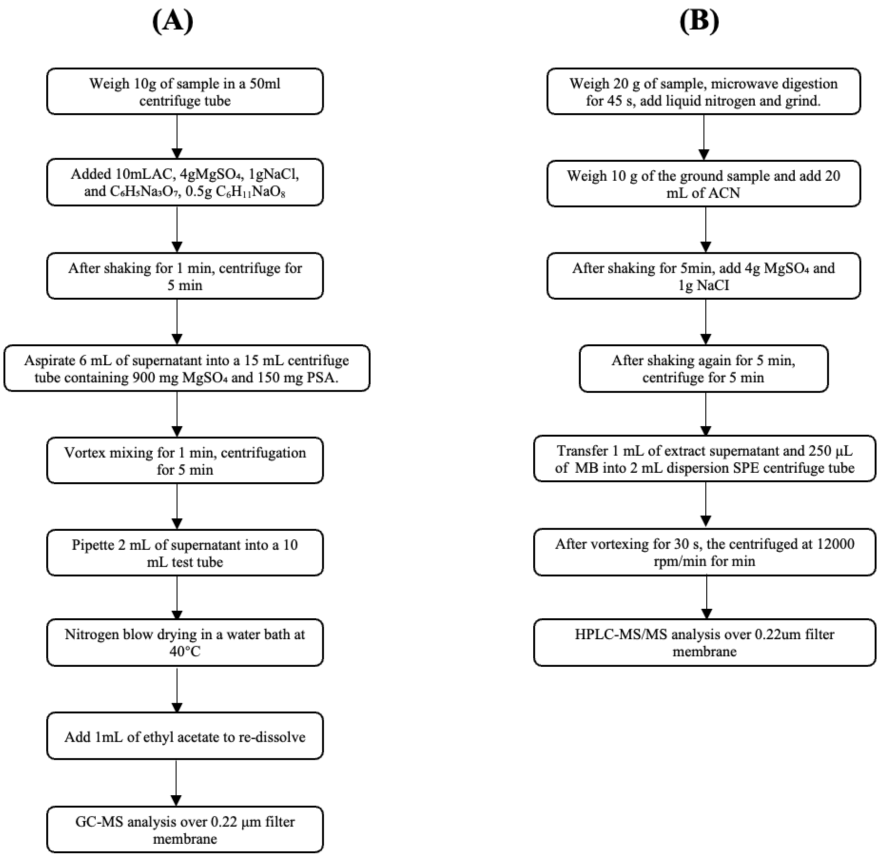

Figure S1. QuEChERS method in GB 23200.113 (A) and the optimized QuEChERS method in this study (B)
